# New approaches to symptomatic treatments for Alzheimer’s disease

**DOI:** 10.1186/s13024-021-00424-9

**Published:** 2021-01-13

**Authors:** Jeffrey Cummings

**Affiliations:** Chambers-Grundy Center for Transformative Neuroscience, Department of Brain Health, School of Integrated Health Sciences, University of Nevada Las Vegas, Las Vegas, NV USA

**Keywords:** Neuropsychiatry, Alzheimer’s disease, Psychosis, Apathy, Agitation, Depression, Drug development, Clinical trials, Cognition

## Abstract

**Background:**

Successful development of agents that improve cognition and behavior in Alzheimer’s disease (AD) is critical to improving the lives of patients manifesting the symptoms of this progressive disorder.

**Discussion:**

There have been no recent approvals of cognitive enhancing agents for AD. There are currently 6 cognitive enhancers in Phase 2 trials and 4 in phase 3. They represent a variety of novel mechanisms. There has been progress in developing new treatments for neuropsychiatric symptoms in AD with advances in treatment of insomnia, psychosis, apathy, and agitation in AD. There are currently 4 AD-related psychotropic agents in Phase 2 trials and 7 in Phase 3 trials. Many novel mechanisms are being explored for the treatment of cognitive and behavioral targets. Progress in trial designs, outcomes measures, and population definitions are improving trial conduct for symptomatic treatment of AD.

**Conclusions:**

Advances in developing new agents for cognitive and behavioral symptoms of AD combined with enhanced trial methods promise to address the unmet needs of patients with AD for improved cognition and amelioration of neuropsychiatric symptoms.

## Background

Alzheimer’s disease (AD) is a progressive neurodegenerative disorder manifested by cognitive and functional decline and the emergence of neuropsychiatric symptoms. The underlying biology of AD includes aggregation of soluble amyloid species into insoluble amyloid plaques, hyperphosphorylation of tau with formation of intracellular neurofibrillary tangles, and neuronal death along with a variety of related processes including neuroinflammation, synaptic and circuit dysfunction, mitochondrial and bioenergetic disorders, epigenetic changes, and vascular abnormalities [1, 2]. Neuronal loss in transmitter system source nuclei leads to neurochemical deficits that contribute to cognitive and behavioral symptoms [3].

All currently approved treatments for AD are “symptomatic” agents that aim to improve cognitive and behavioral symptoms without altering the underlying course of the disease [4]. Most current drug development programs target disease modification with agents that will prevent or delay the onset or slow the progression of AD [5]. No new therapies for AD have been approved by the US Food and Drug Administration (FDA) since 2003 and there are no approved therapies for disease modification of any adult-onset neurodegenerative disorder [5, 6]. Development of symptomatic agents is important; improvement in cognition is a key goal for patients with cognitive impairment, while relief of behavioral symptoms impacts quality of life of patients and caregivers and delays institutionalization. This review summarizes current cognitive enhancing approaches and addresses trends in the development of new symptomatic agents for the treatment of cognitive and behavioral abnormalities of AD. New directions in the treatment of cognitive enhancing agents are presented, and advances in the development of new therapies for neuropsychiatric and behavioral symptoms are discussed.

## Review

### Cognitive enhancing agents for treatment of Alzheimer’s disease

#### Current symptomatic therapies

Four cholinesterase inhibitors (ChE-Is) and one N-methyl-D-aspartate (NMDA) receptor antagonist have been approved by the FDA for the treatment of AD. One ChE-I --- tacrine --- is no longer available on the market; the three available ChE-Is are donepezil (Aricept™), rivastigmine (Exelon™), and galantamine (Razadyne™). The NMDA receptor antagonist is memantine (Namenda™). Clinical trials show that these agents produce improvement on the AD Assessment Scale – cognitive subscale (ADAS-cog) in the range of 1.5 to 3 points (of 70) with corresponding changes on the Mini Mental State Examination (MMSE) [7, 8]. Meta-analyses demonstrate consistent benefit compared to placebo on measures of function and global ratings [9]. Drug-placebo differences persist for a least 1 year in double-blind trials [10]. Cholinesterase inhibitors and memantine have similar effects with improvements above baseline on measures of cognition and global function and temporary stabilization of activities of daily living (ADL). Most studies show amelioration of current neuropsychiatric symptoms with reduced emergence of new neuropsychiatric symptoms following treatment with symptomatic agents [4, 11, 12].

ChE-Is capitalize on the unique biological circumstance in AD of loss of presynaptic cholinergic cells in the nucleus basalis of Meynert with preserved post-synaptic cortical cholinergic neurons, creating the opportunity for functional post-synaptic stimulation producing cholinergic augmentation with corresponding cognitive improvement [13, 14]. The nucleus basalis provides the principal source of acetylcholine for the cerebral cortex and amygdala. Limbic and paralimbic cortices of the brain receive the most cholinergic input and are also the principal sources of reciprocal cortical projections back to the nucleus basalis [15]. No other transmitter system essential to cognition is known to have the pre/post-synaptic disconnection characteristic of the muscarinic cholinergic system, and this may explain in part the many failures to produce cognitive enhancement through stimulation of other transmitter systems.

Cognition is dependent on intact circuit function that includes cholinergic innervation as well as involvement of other transmitters. Imaging investigations such as those with functional magnetic resonance imaging (fMRI) and fluorodeoxyglucose (FDG) positron emission tomography (PET) typically show enhanced cortical circuit activity following treatment with ChE-Is [16, 17]. Enhancement of circuit function is a goal of cognitive-enhancing therapies and new imaging tools may assist in the search for more effective treatments [18].

#### Clinical trial methodology for symptomatic cognitive enhancing agents

The clinical methodology and instrumentation for AD trials was defined by trials of tacrine, the first agent approved for the treatment of AD by the FDA [19, 20]. Patients were selected using the MMSE [21], and outcomes included the Clinical Global Impression of Change (CGIC) [22] and the ADAS-cog [23]. The approval process was based on draft guidelines from the FDA requiring that antidementia agents show improvement on dual outcomes: 1) the core symptoms of AD --- memory and cognition --- and; 2) a global or a functional rating establishing that the effect was clinically meaningful [24]. This approach to AD clinical trials remains highly influential for trials of both putative cognitive enhancing and disease modifying therapies (DMTs). In most current trials, participants are defined by MMSE score range; the ADAS – cognitive subscale (ADAS-cog) is a commonly used outcome instrument in clinical trials for patients with AD dementia; and the CGIC or modified versions of the instrument are used in most trials of cognitive enhancing and behavioral agents being developed for AD. Nearly all trials of cognitive enhancing agents in AD dementia have used the “tacrine formula” for clinical trials with limited implementation of new instruments and measures in trials of patients with mild-moderate AD.

#### Overview of recent trials for cognitive enhancing agents

**Fig. 1:**
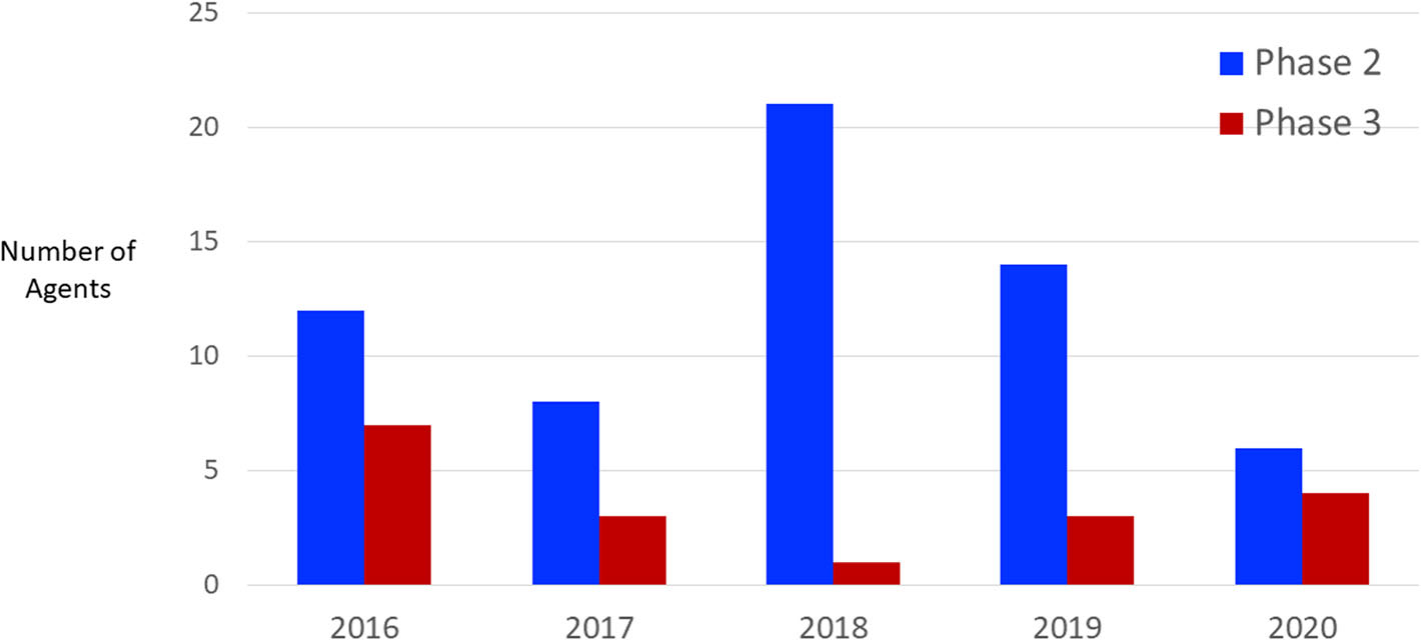
Number of cognitive enhancing agents for Alzheimer’s disease in Phase 2 and Phase 3 clinical trials 2016–2020 [5, 25–28]

Figure 1 shows the clinical trial activity for cognitive enhancing agents from 2016 to 2020. Phase 2 and Phase 3 are shown. The data are derived from annual reviews of the AD drug development pipeline based on clinicaltrials.gov [5, 25–28]. There are more agents in Phase 2 than in Phase 3 for all years. Many agents are not advanced to Phase 3 after a failure to show a drug-placebo difference in Phase 2. There are fewer agents in Phase 3 in 2020 than in any previous year; the number of agents in Phase 2 varies and does not predict the number of agents in Phase 3 at later time points.

#### Cognitive enhancing drugs with novel mechanisms of action in clinical trials

GV-971 (Oligomannate™) is an oligosaccharide that has putative effects on the gut microbiome to reduce systemic inflammation and neuroinflammation [29]. In a 9 month trial conducted in China, participants evidenced a significant improvement above baseline on the ADAS-cog and showed a trend for improvement on the Clinical Interview Based Impression of Change with caregiver input (CIBIC+). These observations were sufficient for approval by the National Medical Products Administration (NMPA; Chinese equivalent of the FDA). GV-971 is the first drug treatment for AD approved anywhere in the world since 2003. The trial did not include biomarkers that might provide insight into the mechanism(s) of action of GV-971. The agent is being assessed in a global Phase 3 trial with clinical measures and biomarker outcomes to assess symptomatic and disease-modifying effects. Neuroinflammation is increasingly recognized as a key aspect of the pathogenesis of AD and an important target for drug development [30]. Dysbiosis of the gut microbiome in AD leads to local and systemic inflammation, release of neuroactive products that promote neuroinflammation, and perturbation of the normal gut-brain axis [31]. GV-971 reverses gut dysbiosis and reduces systemic inflammation in experimental AD model animals [19].

5-HT_6_ antagonists have been the subject of several recent clinical trials. Intepirdine, idalopirdine, and masuperdine are drugs in this class assessed in Phase 3 development programs [32]. All trials have failed to establish a drug-placebo difference on cognitive outcomes. Secondary behavioral outcomes in the masuperdine trial suggested a benefit on agitation and further assessment of the behavioral effects of this agent will be pursued. 5-HT_6_ receptors are exclusively found in the central nervous system where they modulate primarily gamma-aminobutyric acid (GABA) and glutamate levels, facilitating the secondary release of other neurotransmitters including noradrenaline and acetylcholine which are compromised in AD [33].

Cholinergic stimulation can be achieved with nicotinic cholinergic agonists or muscarinic cholinergic agonists in addition to the currently approved ChE-Is. Although promising results have been observed in early stage trials with these agents, none have succeeded in late stage trials or been approved by the FDA [34]. A nicotine transdermal patch is currently in a Phase 3 trial. Nicotinic receptors (α4β2 subtype) are reduced in AD as demonstrated by the receptor-specific PET ligand 18F-flubatine [35].

New versions of ChE-Is are in development programs including octohydroaminoacridine and AD-35 [5].

ChE-Is in combination with peripheral cholinergic receptor blockers have been tested in an attempt to achieve greater central cholinergic stimulation while limiting peripheral cholinergic side effects. CPC-201 is an example of this strategy [36]. These approaches capitalize on the validated cognitive improvements demonstrated with ChE-Is.

A trial of MK-7622, an M1 positive muscarinic allosteric modulator, was stopped for futility and showed no drug-placebo differences on cognitive or functional outcomes [37]. Other members of this class may be explored given the evidence supporting cognitive and behavioral effects of these agents as well as a possible effects on the underlying pathobiology of AD [38, 39].

The adrenergic system is a target of putative cognitive enhancing agents. Guanfacine is an alpha-2 adrenergic agonist currently in trials. The locus coeruleus and associated adrenergic projections to cerebral cortical regions mediating key aspects of cognition and behavior are atrophic in AD making adrenergic function a candidate for AD therapy [40].

The dopaminergic system may play a role in cognitive function, especially executive measures mediated by frontal-subcortical systems. The dopaminergic midbrain is altered in AD and substantial evidence links this structure to the cognitive and behavioral changes of AD [41]. Rotigotine, a dopamine agonist approved for the treatment of motor symptoms of Parkinson’s disease, showed a benefit on frontal-executive measures in a Phase 2 trial for AD [42]. Rasagiline, a monoamine oxidase inhibitor approved for motor symptoms of Parkinson’s disease, improved metabolism on FDG PET in an AD trial and produced benefit on some executive measures and a quality of life scale without improving performance on memory measures [43]. Ladostigil, a multi-target agent with ChE-I and monoamine oxidase inhibition properties, did not improve cognition or delay decline in a Phase 2 trial [44]. Sembragiline, another monoamine oxidase inhibitor, showed no drug-placebo difference in a 12 month Phase 2 trial; patients on active treatment had reduced behavioral symptoms compared to those on placebo [45].

Phosphodiesterase inhibitors (PDE-Is) are another class of agents which have been assessed in multiple AD clinical trials, so far without successfully demonstrating a drug-placebo difference in cognition (Table 1). In many cases, the phosphodiesterase inhibitors have multiple targets with cognitive enhancement being among the potential outcomes and disease modification another potential treatment benefit. Inhibitors of PDE 3, 4, 5 and 9A have been assessed in clinical trials; some PDE inhibitors remain in trials and may ultimately demonstrate a significant cognitive benefit. Phosphodiesterase inhibitors enhance cyclic adenosine monophosphate (cAMP) and/or cyclic guanosine monophosphate (cGMP) signaling by inhibiting degradation of these cyclic nucleotides. cAMP and cGMP signaling are essential in a variety of cellular functions, including neuroplasticity and neuroprotection, and PDEs are increasingly receiving attention as possible targets for treatment of AD [46]. cGMP can be measured in cerebrospinal fluid (CSF) as a target engagement biomarker in human trials of some PDE-Is [47].

**Table 1:** Phosphodiesterase inhibitors assessed in clinical trials (2016–2020) [5, 25–28] (Year refers to the years in which the agent is listed on clinicaltrials.gov as being in a clinical trial)

Year	Agent	PDE Target	Phase	Outcome
2020, 2019	BPN14770	4	2	Synaptic, neuroprotection, anti-inflammatory
2020, 2019, 2018, 2017, 2016	Cilostazol	3	2	Synaptic plasticity and neuroprotection; improved circulation
2019	AR1001	5	2	Synaptic plasticity and amyloid reduction
2017, 2016	BI 409306	9A	3	Cognitive enhancer

Xanamem®(UE2343) is a an 11-ß-hydroxysteroid dehydrogenase (HSD) antagonist intended to produce cognitive enhancement through blocking the adverse effects of endogenous glucocorticoids on memory and cognition [48]. A recent phase 2 trial failed to establish a drug-placebo difference. A similar agent --- ABT-384 --- was terminated for futility when it failed to produce cognitive benefit [49]. Endogenous glucocorticoids have a wide spectrum of physiological effects and are elevated in AD where they have been correlated with dysregulation of the hypothalamic-pituitary-adrenocortical axis, hippocampal degeneration, and reduced cognitive function. Non-clinical studies show that increased glucocorticoids levels accelerate the formation of Aβ in animal models of AD, promoting the amyloidogenic pathway and reducing Aβ clearance [48]. These clinical and nonclinical observations support glucocorticoid reduction as a treatment target in AD.

#### Cognitive enhancing drugs with novel mechanisms of action in non-clinical development

Beyond the mechanisms noted above and addressed in late stage trials, a number of other mechanisms are being considered to achieve cognitive enhancement in AD. Among the targets currently addressed by agents under study are drug-induced effects on metabotropic glutamate receptor 5, cyclic adenosine monophosphate, norepinephrine, glucagon-like peptide 1, polyphenols, flavonoids, D1 and D2 dopamine receptors, and calcium channel modulation [50–55]. Some agents such as calcium channel blockers and oligomannate (described above) may have both cognitive enhancing and disease-modifying properties.

#### Comparison of drug development for cognitive enhancing and disease-modifying treatments for Alzheimer’s disease

There are substantial challenges in the development of both DMTs and cognitive enhancing agents. Table 2 presents the comparative advantages and challenges to be considered for each type of development program. Symptomatic therapies have an established regulatory pathway, known trial methodology with widely accepted outcome measures (primary and secondary), targets that are relatively well understood (ion channels and transmitter receptors), trials that demonstrate improvement above baseline and require relatively smaller sample sizes and shorter durations of exposure to observe drug-placebo differences, can be administered conveniently as oral medications, and usually cost less to develop. DMTs must establish a regulatory pathway and identify a validated target, require larger and longer trials, are more costly to develop, are expected to be less convenient (intravenous or subcutaneous administration in many cases such as monoclonal antibodies), and will be more costly for patients and healthcare systems. Unique features of DMT development include possible extension to asymptomatic preclinical populations; use of biomarkers to select patients, show target engagement, and support disease-modification; and expectation of cumulative benefit and increasing drug-placebo difference with continued adherence to the medication regimen. Both types of therapy are needed to improve outcomes in individuals with AD continuum disorders, and it is highly likely that DMT and symptomatic therapies will be administered simultaneously to optimize patient benefit. Most current trials allow participants to be on the standard of care with ChE-Is with or without mementine; participants are randomized to drug or placebo on the background of approved therapy. This is one paradigm for combination therapy when an approved agent is allowed in a trial for a test agent. Sponsors and developers need to anticipate these challenges when considering development programs for DMTs or symptomatic therapies.

**Table 2:** Development of symptomatic cognitive enhancing drugs compared to development of disease-modifying agents

Development	Symptomatic Cognitive Enhancing Agent	Disease-Modifying Agent
Approved agents	Yes	No
Defined regulatory pathway	Yes	No
Validated target	Yes	No
Conventional target	Yes; receptors and ion channels	Some conventional targets such as enzymes and some unconventional targets such as protein-protein interactions
Clinical outcomes validated in successful trials	Yes	No
Trial sample size	200–400 per arm	600–1200 per arm
Trial duration	3–6 months	12–24 months
Administration	Oral; patch	Oral; intravenous; subcutaneous; intramuscular
Patient convenience	More convenient; usually orally administered	Less convenient if intravenous, subcutaneous, or intramuscular administration required
Diagnosis	Usually phenotype-based	Phenotype-based usually with biomarker confirmation
Target engagement biomarker	May not be included	Yes
Disease-modification biomarker	No	Yes
Preclinical application	No; symptoms required	Yes; trials can be biomarker based
Prodromal application	Yes; none approved	Yes; none approved
Alzheimer dementia application	Yes; approved	Yes, none approved
Increasing drug-placebo difference over time	No; cumulative benefit not anticipated	Yes; cumulative benefit anticipated
Delayed start or randomized withdrawal trial design show enduring effect of treatment	No	Yes
Cost of development program	Lower	Higher
Cost of treatment to patients	Lower	Higher
Patient message	Improved ability above baseline function	No improvement; less decline over time; delayed onset of dementia (in predementia populations)

The contrast of symptomatic cognitive enhancing therapies and DMTs artificially simplifies these categories. There is evidence that combination therapy with cholinesterase inhibitors and memantine slows the progression of AD and may have a disease modifying effect [56]. Some cholinesterase inhibitors and M1 muscarinic agonists reduce amyloid precursor protein (APP) processing and lower amyloid plaque and neurofibrillary tangles in cell and animal models, respectively [57, 58]. Agents considered DMTs may have symptomatic effects through rescue of dysfunctional but not moribund cell populations by reducing local toxicity and enhancing neuroprotection [59, 60]. Thus, DMTs can plausibly produce cognitive enhancement, and symptomatic therapies may have mechanistic properties that produce disease modification. In addition, DMTs may exhibit psychotropic properties by suppressing emergence of new behavioral symptoms if they effectively reduce the rate of progression of AD [61]. The conventional terminology derives from the developmental intent to interrupt cell death in the case of DMTs or to enhance cognition or reduce behavioral alterations with the symptomatic agents.

### Treatment of neuropsychiatric symptoms of Alzheimer’s disease

#### Overview of recent trials for neuropsychiatric symptoms of Alzheimer’s disease

**Fig. 2:**
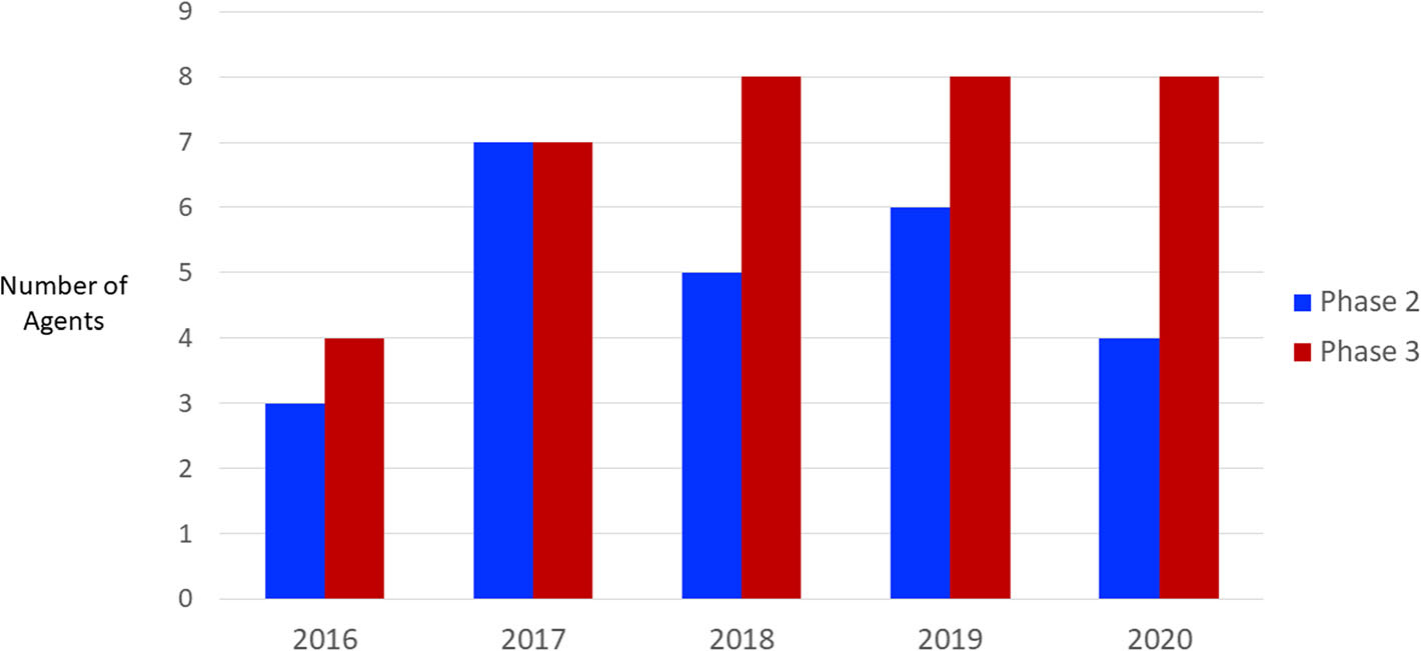
Number of agents addressing neuropsychiatric symptoms of Alzheimer’s disease in Phase 2 and Phase 3 clinical trials 2016–2020 [5, 25–28]

There are currently no approved treatments for any neuropsychiatric symptom in AD; there is progress in clinical trials and trial methodology, several drugs are in late stage trials for behavioral disorders in AD or dementia, and one agent has been submitted to the FDA for approval as treatment for dementia-related psychosis (discussed below). Most of the agents in trials are re-positioned from successful development programs for behavioral changes in patients with major mental disorders (e.g., schizophrenia, bipolar illness, major depressive disorder, insomnia). Figure 2 shows the number of drugs in clinical trials for the years 2016–2020 that addressed behavioral symptoms as the primary outcome of the trials [5, 25–28]. The number of agents is relatively small; 4–8 in Phase 3 and 2–7 in Phase 2, over the 5 year period. The trials have been facilitated by development of consensus syndrome definitions useful in clinical trials including agitation in cognitive impairment [62], psychosis in major and mild neurocognitive disorders [63], and apathy in dementia [64].

The neurobiological bases of behavioral changes in AD are not precisely known. Behavior is an emergent property of integrated circuits with distributed anatomies and reliance on multiple transmitters. Distinct circuits comprising unique anatomies and neurotransmitters underly different behavioral phenotypes [65]. In AD, the presence of a variety of types of neurotoxic protein assemblies (amyloid, tau, alpha-synuclein, transactive response DNA binding protein-43 [TDP-43]) induce synaptic dysfunction and network/circuit dysfunction [66]. Circuit abnormalities give rise to behavioral syndromes with neurobiologic, genetic, and resilience factors all contributing to the final behavioral phenotype exhibited by the patient. Measures such as FDG metabolism reflect synaptic dysfunction and demonstrate distinct behavior-related circuit patterns including right frontotemporal hypometabolism in patients with agitation [67]; bilateral anterior cingulate and orbitofrontal hypometabolism in patients with apathy [68]; or decreased orbitofrontal metabolism in AD patients with psychosis [69]. Exploration of specific pathologic and biochemical correlates of behavioral changes in AD has been hampered by the long period between when the patients come to autopsy and the time the behavioral symptoms are manifest [70].

#### Agitation

Development of new therapies for agitation is currently the most active area of drug development for neuropsychiatric symptoms in AD. There are 8 agents in clinicals trials for agitation. Brexpiprazole (Rexulti™) is approved for treatment of schizophrenia and as adjunctive therapy for major depressive disorder. Preliminary trials in AD demonstrated a drug-placebo difference in agitation reduction with the 2 mg daily dose, not the 1 mg dose [71]. A confirmatory trial is in progress. Dextromethorphan/quinidine (DM/Q. Nuedexta™) was shown to reduce agitation in a Phase 2 trial [72]. Phase 3 trials of the deuterated version of DM/Q failed to confirm the anti-agitation effect, and additional trials are on-going. AX-05, a combination of dextromethorphan and bupropion, had a successful Phase 2 trial with a significant drug-placebo difference in agitation reduction. Phase 3 trials are planned. Nobilone, a cannabinoid agent, was shown to reduce agitation in AD in a double-blind crossover study [73]. Sedation was more common in the nobilone-treated patients compared to those receiving placebo. Prazosin, a postsynaptic alpha-1 adrenergic receptor antagonist has shown preliminary anti-agitation effects and is being further assessed [74].

#### Psychosis

There are no approved treatments for psychosis in any form of dementia. Pimavanserin (Nuplazid™) is approved for psychosis in Parkinson’ disease (PD) without dementia [75]. Pimavanserin was shown to be effective in a Phase 2 trial of AD with psychosis [76]. These development milestones led to the HARMONY trial of dementia-related psychosis [77]. This was a randomized discontinuation trial that includes participants with five types of dementia – AD, Parkinson’ disease dementia, dementia with Lewy bodies, frontotemporal lobar degeneration, and vascular dementia. The trial met its primary outcome of earlier relapse in the placebo group compared to the active therapy group, and pimavanserin has been submitted for possible marketing approval by the FDA.

Psychosis is associated with more rapid cognitive and functional decline in AD [78, 79]. Future studies will determine if successful treatment of psychosis will ameliorate the rate of cognitive and functional loss.

#### Apathy

Apathy is among the most common manifestations of AD and other dementias [80]. No treatment has been approved by the FDA for treatment of this neuropsychiatric syndrome. Methylphenidate (Ritalin™) was assessed in a Phase 2 trial and demonstrated improvement on the Clinical Global Impression of Improvement (CGI-I) and the apathy subscale of the Neuropsychiatric Inventory (NPI) [81]; the drug-placebo difference on the Apathy Evaluation Scale (the primary outcome of the trial) was not significant [82]. A follow-up study is in progress [83]. A clinical trial of modafinil in apathy did not establish a drug placebo difference [84].

PET and fMRI studies of apathy in neurodegenerative disorders show abnormalities in frontal regions associated with impairments in planning and decision making and in the anterior cingulate cortices related to emotional blunting and loss of motivation [85].

#### Sleep

Sleep disorders are common in AD and affect the lives of the patient and the caregiver [86, 87]. Poor sleep adversely impacts cognition and behavior. In addition, there is increasing evidence that sleep deprivation increases the concentration of soluble amyloid in the brain and results in accumulation of insoluble amyloid; sleep extension has the opposite effect [88]. Sleep deprivation also increases levels of the tau protein in human CSF and accelerates the spread of tau aggregates in neural networks [89].

Suvorexant, a dual orexin antagonist (DORA), was recently shown in a double blind, placebo-controlled phase 3 trial to reduce insomnia in AD. Total sleep time was significantly increased and wakefulness after sleep onset (WASO) was significantly decreased [90]. Suvorexant had a prior approval for insomnia and successful treatment of insomnia in AD did not result in specific labeling for insomnia in AD. However, clinical and adverse event information is now included in the Package Insert to guide practitioner use of suvorexant in AD. This highlights the challenge of pseudospecificity in labeling for AD behavioral indications when the agent tested for treatment of AD has been approved for a similar indication in a non-AD disorder [24].

Lemborexant, a DORA, is being investigated for irregular sleep wake rhythm disorder (ISWRD) in AD [91]. ISWRD is a circadian rhythm disorder characteristic of AD, other NDD, and some developmental disorders. ISWRD is thought to be produced by degeneration of neurons of the suprachiasmatic nucleus (SCN) of the hypothalamus, decreased responsiveness of the circadian “clock” to entraining agents such as light and activity, and decreased exposure to bright light and structured social and physical activity during the day common in cognitively compromised individuals. Treatment of ISWRD seeks to restore a more normal circadian rhythm by consolidating sleep during the night and wakefulness during the day [92]. Restoration of daily circadian rhythms is a novel approach to AD therapy that may have sleep, wakefulness, cognitive, and behavior effects. With appropriate biomarkers and trial designs, such interventions could be demonstrated to be DMTs given the growing evidence of the relationship between sleep disorders and exacerbation of AD pathology [88, 93].

Additional agents currently under study for sleep disorders in AD include zopiclone and zolpidem [5].

#### Challenges for clinical trial of agents for treatment of neuropsychiatric syndromes

A major challenge for clinical trials of treatments for neuropsychiatric syndromes is the occurrence of robust placebo-group improvement. This reduction in symptoms in the no-active-treatment group may be a true placebo response in the participant, a placebo response in the caregiver who reports the response in most clinical trials, regression to the mean of elevated scale scores at the time of screening for the trial, natural history of agitation, and behavioral improvement in response to the clinical trial circumstances including increased family involvement, greater staff attention in residential settings, and interaction with research staff [94].

The substantial placebo response in behavioral trials is the greatest challenge to being able to demonstrate a treatment effect of the active agent. A variety of strategies have been implemented in an attempt to understand and manage the placebo-group effects in trials of neuropsychiatric agents (Table 3). Placebo run-in periods are used in randomized clinical trials to exclude patients after screening, but before randomization. In theory, run-in periods increase the probability of detecting a potential treatment effect, although they may reduce both external and internal validity [95]. Placebo run-in periods help identify patients who respond to the placebo in the early period of therapy, and these can be excluded from the trial if they no longer meet entry criteria. Run-in periods help to examine participant adherence, and those unlikely to comply with study procedures and whose inclusion would reduce study power can be excluded at the time of randomization [96]. A variant of the placebo run-in was used in the pimavanserin trial for Parkinson’s disease psychosis [75]. Patients received 2 weeks of psychosocial therapy prior to randomization and those responding and no longer meeting criteria for study entry were excluded. Fifty-three patients (40% of excluded patients, 17% of screened patients) did not meet study entry criteria at the time of randomization although they had appropriate scores at the time of screening. If no placebo run-in is conducted, there is still a period between screening and randomization that may affect participant scores. Participants should meet study criteria at screening and at randomization to limit the effects of regression to the mean or early response to study participation [97]. Patients with more severe symptoms tend to have more evident responses to therapy, and substantial sustained psychopathology should be required for entry [76]. Across trials it is observed that those with 2 arms and a 1:1 randomization to drug or placebo have smaller placebo responses (and larger drug-placebo differences) than multi-arm trials [98]. Central review of the collection of the primary outcomes measures and the entry criteria (at randomization) helps ensure that the behavioral changes are accurately rated and sufficiently severe to meet the randomization criteria [99]. Central reviewers listen to a recorded audio tape of the interview or may watch a videotaped interview. Competent central reviewers must be identified for each language in which the interviews are conducted. The sequential parallel comparison design (SPCD) was created specifically to provide insight into the placebo response. The SPCD has 2 stages: stage 1 is a typical placebo-controlled design; stage 2 entails the re-randomization of placebo nonresponders (patients in the placebo arm of stage 1 who did not improve) to drug or placebo [100]. This design in used in depression and pain trials; the dextromethorphan/quinidine trial of agitation in AD is the only example of its application in AD [72]. In this trial, there was a drug-placebo separation in both stage 1 and stage 2. The randomized discontinuation design is another strategy for limiting placebo responses. All patients are treated in the first period of the trial with the active therapy. Responders to therapy are then randomly assigned to drug or placebo; the primary outcome is the rate of symptom relapse or time to symptom relapse in the placebo arm compared to the active therapy arm. The design selects for those most likely to respond and minimizes placebo responses. Randomized discontinuation trials have demonstrated the efficacy of haloperidol, risperidone, and pimavanserin for behavioral disturbances in AD [77, 101, 102].

**Table 3:** Design adjustments used to manage and understand placebo effects in trials for neuropsychiatric symptoms and syndromes

Design Adjustment	Purpose
Placebo lead-in	Exclude patients who no longer meet trial entry criteria after 2 weeks of placebo treatment Identify non-adherent patients whose participation in the trial would reduce power to observe a drug-placebo difference
Pre-randomization psychosocial intervention	Exclude patients who no longer meet trial entry criteria after 1–2 weeks of psychosocial treatment
Participants meet entry criteria at screening and baseline	Reduce chance of score improvement by regression to the mean
Central review of scales whose scores determine entry to the trial	More reliable review of the data with fewer site influences
Patients have at least moderately severe symptoms at screening and randomization	More severe symptoms are less likely to respond to placebo
Longer trials of 12 to 24 weeks	Placebo responses are often greatest at study onset and become gradually less marked
Two-arm design with 1:1 randomization	Placebo responses are higher in trials with several active treatment arms
Sequential parallel comparison design (SPCD)	2nd stage of the SPCD has only placebo non-responders in the placebo (and active) arm of the trials
Randomized discontinuation design	All participants are on active treatment in the first period of the trial; only responders to active therapy are randomized to drug or placebo

#### Drugs for treatment of neuropsychiatric symptoms with novel mechanisms of action in non-clinical development

Most drugs directed at behavioral symptoms in AD are assessed first in primary psychiatric disorders such as schizophrenia, bipolar illness, depression, or sleep disorders. The absence of a known symptom-specific biology for the neuropsychiatric syndromes of AD makes it difficult to target these behaviors with agents unique to AD. There are few neuropsychiatric agents in Phase 1 AD development programs because the early evaluation of the agents is conducted in psychiatric programs. The diversification of targets for schizophrenia suggests that agents with more varied mechanisms of action will eventually be directed to behavioral symptoms of AD. Glutamatergic agents (metabotropic 1, 2, and 3 receptor agonists), serotonin agents (5-HT_1A_ agonists, 5-HT_2C_ antagonists and agonists, 5-HT_3_ antagonists, 5-HT_6_ antagonists, and 5-HT_7_ antagonists), gamma-aminobutyric acid (GABA) allosteric modulators, neuropeptide agents (neurokinin-3, neurotensin), cannabinoids, anti-inflammatory agents (producing cytokine reduction), and targeting of trace amine-associated receptor 1 (TAAR1) and 5-HT_1A_ are being assessed in nonclinical models of schizophrenia or in early human trials and, if successful, will likely be evaluated for their ability to ameliorate psychosis or agitation in AD [103, 104].

There are no drugs currently in clinical trials for treatment of depression in AD despite a high prevalence of depressive mood changes in the disorder [105]. Conventional antidepressants approved for treatment of major depression have largely failed in clinical trials for depression of AD [106]. Antidepressants with novel mechanisms being assessed for depression in major depression and bipolar disorder can be expected to be evaluated for treatment of depression in AD. Approaches being assessed for depression include targeting inflammatory cytokines (TNF- *α*, IL-1B, IL-6), oxidative and nitrosative stress, PPAR *γ*, glucagon-like peptide 1, mitochondrial modulation and bioenergetics, glutamatergic pathways, S-adenosyl-methionine, neurotrophin signaling, PDE 10A, receptors of psychedelic agents, and the opioid system [107–111].

## Discussion

There is a high rate of negative outcomes in clinical trials for drugs being developed to improve cognitive and behavioral symptoms of AD [6]. Most trials for symptomatic agents do not confirm the diagnosis of AD with biomarkers and use the AD amnestic phenotype for trial inclusion. In the PRIME trial of aducanumab it was shown that 39% of patients with the mild AD dementia phenotype did not have brain amyloid when assessed with amyloid PET [112]. This suggests that a substantial number of participants in trials for symptomatic agents do not have the underlying biology of AD. These agents are not amyloid-focused or amyloid-dependent but including patients with non-AD disorders makes it more difficult to extrapolate results from AD animal models to human trials, may reduce the power of the trial to demonstrate a drug-placebo difference, may result in slower progression in the placebo group, and impedes the developer’s ability to understand the relationship of the intervention to the underlying biology. The evolution of blood tests that accurately predict brain amyloidosis and other AD pathology may greatly improve the feasibility of creating more homogeneous trial populations for assessment of symptomatic agents [113–115].

Precision drug development depends on demonstrating a pharmacodynamic effect of the agent that predicts the expected cognitive or behavioral impact [116]. A variety of such pharmacodynamic biomarkers are evolving for use in trials of DMTs; few have been developed for symptomatic agents. CSF cGMP is increased in trials of PDE inhibitors and provides a useful pharmacodynamic readout [117]. FDG PET is a measure of synaptic function, and the hypometabolism observed in AD is partially reversed by symptomatic agents such as ChE-Is that affect synaptic function [118]. FDG PET showed less reduction of metabolism in response to the monoamine oxidase inhibitor rasagiline compared to placebo supporting a pharmacodynamic effect of this agent [43]. Similarly, cerebral blood flow is increased by symptomatic agents as measured by PET [119]. fMRI demonstrates changes in connectivity following cholinergic therapy [120] and may be useful in assessing other classes of symptomatic agents. More pharmacodynamic measures are needed to inform development of symptomatic agents and to improve the translational gap that exits between nonclinical observations and human responses.

Sensitive clinical outcomes are needed to demonstrate drug-placebo differences, and new tools that promise to perform well in trials are being introduced. The Neuropsychological Test Battery (NTB) may be more sensitive than the ADAS-cog in mild AD and more likely to show a drug-placebo difference in this population where progression is modest in the course of a short trial [121]. Computerized assessments have advantages in standardized administration, reduction of missing data, collection of variables such as reaction time not available from paper-and-pencil tests, and automated interface with electronic databases of trial sponsors [122]. These assessments may be particularly useful in Phase 2 trials with smaller sample sizes and an experimental medicine approach. Matching the cognitive signature of an agent’s mechanism of action to the target pathology or transmitter change may improve trial outcomes. A trial of rotigotine --- a dopaminergic agent influencing frontal-subcortical circuits --- had no effect on the ADAS-cog but showed a significant improvement on the Frontal Assessment Battery (FAB) [42]. The FAB assesses executive/frontal function; the ADAS-cog lacks executive/frontal measures. The Amsterdam-Instrumental Activities of Daily Living Scale [123] may better characterize ADLs of contemporary life and allow more reliable differentiation between drug and placebo than previous instruments. Cognitive-functional composites may be more sensitive, reliable, and more accurate in reflecting patient status and capturing longitudinal change critical to successful trials [124, 125]. Composites are being explored in trials of patients with mild cognitive impairment and early AD dementia.

This review is limited by its dependence on clinicaltrials.gov as the source of information on agents currently in trials. While this registry is comprehensive, it under-represents agents in Phase 1 and some cognitive enhancers or psychotropic agents in early development may have escaped detection. There is no similar registry for agents for behavioral and cognitive symptoms of AD in the nonclinical phases of development, and the catalogue of agents in this stage is no doubt flawed. The principles of drug development applicable across development programs are emphasized.

## Conclusions

There has been progress in developing new therapies for behavioral aspects of AD. Suvorexant has been shown to improve insomnia in AD, and pimavanserin has been submitted to the FDA for dementia-related psychosis including psychosis in AD. Trials of agitation and apathy have promising preliminary results. New criteria for specific behavioral syndromes (agitation, psychosis, apathy) in AD assist in reducing heterogeneity of trial populations, presenting agents to the FDA, and educating prescribers on how best to use emerging treatments. Trial designs and means of managing the placebo response in trials of behavioral disorders have advanced. Less progress is currently evident in the development of cognitive enhancing agents but better trial participant characterization, use of pharmacodynamic biomarkers, and implementation of more sensitive outcomes may assist in improving the success of development programs. Continued improvements will result in progress toward developing cognitive enhancing and psychotropic agents to address the unmet needs of patients with AD.

## Data Availability

Not applicable.

## Abbreviations

Aβ: Amyloid beta
AD: Alzheimer’s disease
ADAS: Alzheimer’s Disease Assessment Scale
ADAS-cog: Alzheimer’s Disease Assessment Scale – cognitive subscale
ADL: Activities of daily living
cAMP: Cyclic adenosine monophosphate
CGIC: Clinical Global Impression of Change
CGI-I: Clinical Global Impression of Improvement
cGMP: Cyclic guanosine monophosphate
ChE-Is: Cholinesterase inhibitors
CIBIC+: Clinical Interview Based Impression of Change with caregiver input
CSF: Cerebrospinal fluid
DM/Q: Dextramethorphan/quinidine (Nuedexta™)
DMT: Disease-modifying therapy
DORA: Dual orexin antagonist
FAB: Frontal Assessment Battery
FDA: U.S. Food and Drug Administration
FDG: Fluorodeoxyglucose
fMRI: Functional magnetic resonance imaging
GABA: Gamma-aminobutyric acid
GV-971: Oligomannate™
HSD: Hydroxysteroid dehydrogenase
ISWRD: Irregular sleep wake rhythm disorder
MMSE: Mini-Mental State Examination
NDD: Neurodegenerative disorder
NMDA: N-methyl-D-aspartate
NMPA: National Medical Products Administration
NPI: Neuropsychiatric Inventory
NTB: Neuropsychological Test Battery
PD: Parkinson’s disease
PDE: Phosphodiesterase
PDE-Is: Phosphodiesterase inhibitors
PET: Positron emission tomography
SPCD: Sequential parallel comparison design
TAAR1: Trace amine-associated receptor 1
WASO: Wakefulness after sleep onset
