# Correction to: New approaches to symptomatic treatments for Alzheimer’s disease

**DOI:** 10.1186/s13024-021-00446-3

**Published:** 2021-04-01

**Authors:** Jeffrey Cummings

**Affiliations:** Department of Brain Health, School of Integrated Health Sciences, University of Nevada Las Vegas, Las Vegas, NV USA

**Correction to: Mol Neurodegeneration 16, 2 (2021)**

**https://doi.org/10.1186/s13024-021-00424-9**

The original article [1] contains an error in the spelling of Masupirdine.

All instances of ‘Masuperdine’ should thus be considered as ‘Masupirdine’.
